# Recurrence of Decay

**Published:** 1891-06

**Authors:** F. S. Whitslar


### Recurrence of Decay.

By F. S. Whitslar.

Read Before the Northern Ohio Dental Association.

There is no topic in the realm of dentistry that has continued to excite the scientific interest of dentists more than caries or decay of the teeth. The different theories which have been held and promulgated concerning the origin of dental decay, proves that the problem is no easy one. Among the more important causes which have been assigned for the decay of the teeth, we note the following:

Inflammation, eloctrolytic decomposition, chemical dissolution, disturbances of nutrition, chemico-parasitic influences.

Bödecker, Heitzmann and Frank Abbot, in their contributions to dental literature, have strenuously advocated the inflammatory theory of decay.

Bridgman, whose treaties won the prize offered by the Odontological Society of Great Britain for the best essay on dental decay, advocated the electrical theory of decay.

Berdmore, who in 1771, by experiments, discovered the action of acids on the teeth, advocated the chemical theory of decay, and up to the present time, perhaps no theory of dental decay has had the approbation of so large a number of able and distinguished odontologists as the chemical, of these the following may be named:

Becker, Magitot Wedl, Tomes, Schlenker, Watt and Taft.

PARASITIC THEORY OF DENTAL DECAY.

Of this theory Dr. Sudduth says: "Dr. Miller's theory of the formation of cavities by the action of a digestive ferment upon the basis-substance of dentine, has been the only theory ever advanced that explains the formation of cavities."

Ad. Weil writes: "Decay generally begins from without, and must therefore first make its way through the enamel cuticle. I regard it as highly probable that this fungus (Lepthothrix buccalis) bores directly through it. The fungi now proceed farther into the enamel and force apart its prisms, gradually breaking up its structure."

Dr. Pierce writes: "I am a firm believer that dental caries cannot progress without these low forms of life."

Black maintains that the chemico-parasitic theory, without a reasonable doubt, gives the true explanation of the etiology of dental caries.

Having thus briefly noticed some of the theories advocated, it is worthy of note that all agree that there is the predisposing as well as the exciting cause of dental decay.

As our subject, "Recurrence of Decay," involves a consideration of both, your attention is directed to the following as some of the predisposing causes of recurrence of decay: Inherited idiosyncrasies, faulty nutrition at the time of development, by reason of which the relative proportions of organic and inorganic material needed for an enduring structure fail to be supplied. Crowded and irregular position, broad, grinding surfaces in contact, and broad triangular spaces intervening at the necks of the teeth, which furnish deposists for foreign matter; a want of thoroughness in removing diseased structure in the preparation of cavities, sometimes the result of color-blindness, sometimes to spare the patient the excessive pain accompanying the operation, or perhaps because the patient cannot or will not bear the pain. Sometimes for the sake of our backs we decide that a difficult cavity is ready to fill, when a careful examination of it, after wiping out the cavity with a pledget of cotton or spunk saturated with carbolic acid, might still reveal soft points. Improperly shaped margins, especially at the cervical border, fractured walls, excessive malleting on teeth of soft structure, overlapping and rough margins of fillings, are some of the predisposing causes of recurrence of decay, some of which invite the action of exciting causes of decay. Another cause of recurrence of decay is a lack of repeated, thorough, systematic cleansing of the oral cavity and the teeth, thereby reducing the amount of fermentable substances as to materially diminish the production of acid, which is most active in the nascent state.

